# Bis[μ-2-(2*H*-benzotriazol-2-yl)-4-methyl­phenolato]bis­[dimethyl­aluminium(III)]

**DOI:** 10.1107/S1600536809018492

**Published:** 2009-05-23

**Authors:** Chen-Yu Li, Chia-Her Lin, Bao-Tsan Ko

**Affiliations:** a Department of Chemistry, Chung Yuan Christian University, Chung-Li 320, Taiwan

## Abstract

The title complex, [Al_2_(CH_3_)_4_(C_13_H_10_N_3_O)_2_], is dimeric, bridged through the O atoms of the phenolate anions. The asymmetric unit contains one half of the mol­ecule and there is a crystallographic inversion centre in this mol­ecule. Each Al atom is penta­coordinated by one N atom and two bridging O atoms of two *N*,*O*-bidentate benzotriazolylphenolate ligands and by two C atoms from two methyl groups, forming a distorted trigonal–bipyramidal environment.

## Related literature

For background information, see: Liu *et al.* (2001 ▶); Wu *et al.* (2006 ▶). For related structures, see: Lewinski *et al.* (2003 ▶); Tsai *et al.* (2009 ▶).
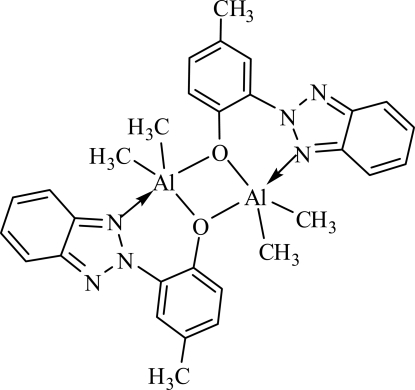


## Experimental

### 

#### Crystal data

[Al_2_(CH_3_)_4_(C_13_H_10_N_3_O)_2_]
*M* *_r_* = 562.58
Triclinic, 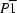

*a* = 7.4220 (4) Å
*b* = 9.7120 (5) Å
*c* = 11.6331 (6) Å
α = 112.517 (2)°
β = 94.824 (3)°
γ = 109.574 (2)°
*V* = 707.79 (7) Å^3^
*Z* = 1
Mo *K*α radiation
μ = 0.14 mm^−1^
*T* = 296 K
0.48 × 0.25 × 0.25 mm

#### Data collection

Bruker APEXII CCD diffractometer
Absorption correction: multi-scan (*SADABS*; Bruker, 2008 ▶) *T*_min_ = 0.937, *T*_max_ = 0.966
11757 measured reflections
3405 independent reflections
2923 reflections with *I* > 2σ(*I*)
*R*_int_ = 0.017

#### Refinement

*R*[*F* ^2^ > 2σ(*F* ^2^)] = 0.039
*wR*(*F* ^2^) = 0.122
*S* = 1.03
3405 reflections
182 parameters
H-atom parameters constrained
Δρ_max_ = 0.28 e Å^−3^
Δρ_min_ = −0.26 e Å^−3^

### 

Data collection: *APEX2* (Bruker, 2008 ▶); cell refinement: *SAINT* (Bruker, 2008 ▶); data reduction: *SAINT*; program(s) used to solve structure: *SHELXS97* (Sheldrick, 2008 ▶); program(s) used to refine structure: *SHELXL97* (Sheldrick, 2008 ▶); molecular graphics: *SHELXTL* (Sheldrick, 2008 ▶); software used to prepare material for publication: *SHELXTL*.

## Supplementary Material

Crystal structure: contains datablocks I, global. DOI: 10.1107/S1600536809018492/rk2147sup1.cif

Structure factors: contains datablocks I. DOI: 10.1107/S1600536809018492/rk2147Isup2.hkl

Additional supplementary materials:  crystallographic information; 3D view; checkCIF report

## Figures and Tables

**Fig. 1.**
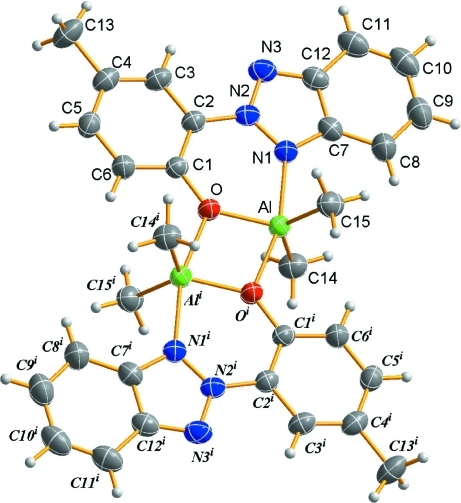
A view of the molecular structure of I with the atom numbering scheme. Displacement ellipsoids are drawn at the 30% probability level. H atoms are presented as a small spheres of arbitrary radius. Symmetry code: (i) -x, -y, -z + 2.

**Fig. 2.**
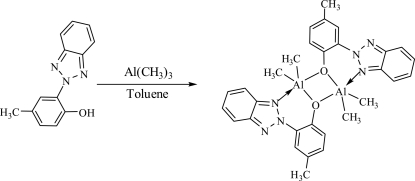
The preparation pass for the title compound I.

## supplementary crystallographic information

### Comment

Due to the biodegradable, biocompatible, and permeable properties, aliphatic polyesters, such as poly(ε-caprolactone) - (*PCL*), poly(lactide) - (*PLA*) and their co-polymers have been widely used in the biomedical and pharmaceutical fields. Therefore, it has been of great interest to develop new catalytic/initiating systems for the preparation of *PCL* and *PLA*. Metal complex-catalyzed ring-opening polymerization (*ROP*) of lactones/lactides has been proven to be the most promising method to synthesize these polymers (Wu *et al.*, 2006). Among them, a variety of main group metal complexes, such as magnesium, zinc, tin, lithium, and calcium as well as aluminum complexes have been reported to be efficient initiators/catalysts. In particular, Liu with co-workers, (2001) have reported the aluminum alkoxide complexes supported by the bulky bisphenol ligand and these complexes have been demonstrated as efficient initiators to catalyze *ROP* of caprolactones and lactides. Recently, our group is interested in the synthesis and preparation of various metal complexes derived from the benzotriazol-phenol ligands. For instance, we have successfully synthesized and structural characterized a Pd(II) complex with 4-methyl-2-(2*H*-benzotriazol-2-yl)-phenolate ligands (Tsai *et al.*, 2009). We report herein the synthesis and crystal structure of *N*,*O*-bidentate benzotriazol-phenolate ligands incorporated Al^III^ complex (I), a potential catalyst for the *ROP* of cyclic esters in the presence of alcohols (Scheme 1).

The solid structure of title compound (I) reveals a dimeric Al^III^ complex (Fig. 1), doubly bridged through the O atoms of the phenolate anions. It was found that the asymmetric unit has one half of molecule and there exists a crystallographic inversion centre of symmetry in this molecule. The geometry around each Al atom is penta-coordinated with a distorted trigonal bipyramidal environment in which one N atom and two bridging O atoms come from *N*,*O*–bidentate benzotriazol-phenolate ligand and two C atoms are from two methyl groups. The sums of bond angles around Al center are 359.97 (7)°. The bond distances between the Al atom and O, N1, O^i^ (symmetry code: (i) -*x*, -*y*, -*z* + 2), C14 and C15 are 1.8338 (11), 2.1061 (13), 2.0918 (11), 1.9767 (17), 1.9725 (17) Å, respectively. These bond distances are longer than those found in other Schiff base Al^III^ complexes with four-coordinated geometry (Lewinski *et al.*, 2003). It is interesting to note that the six-member ring formed from the bidentate benzotriazol-phenolate ligand and Al atom is almost coplanar with the mean deviation of 0.006 (2) Å.

### Experimental

The title compound I was synthesized by the following procedures (see Fig. 2): to a rapidly stirred solution of 4-methyl-2-(2*H*-benzotriazol-2-yl)phenol (0.22 g, 1.0 mmol) in toluene (20 ml) was slowly added Al*Me*_3_ (0.5 ml, 1.0 mmol). The mixture was further stirred at room temperature for 2 h and then dried under vacuum. The residue was extracted with hot toluene (10 ml) and the saturated solution was cooled to 273 K, yielding yellow crystals. Yield: 0.24 g (86%). ^1^H NMR (CDCl_3_, p.p.m.): δ 7.04–8.14 (14H, m, ArH), 2.40 (6H, s, CH_3_), -0.57 (12H, s, AlCH_3_).

### Refinement

The H atoms were placed in idealized positions and constrained to ride on their parent atoms, with C—H = 0.93 Å and 0.96 Å with *U*_iso_(H) = 1.2 and 1.5*U*_eq_(C).

### Crystal data

**Table:**

[Al_2_(CH_3_)_4_(C_13_H_10_N_3_O)_2_]	*Z* = 1
*M**_r_* = 562.58	*F*(000) = 296
Triclinic, *P*1	*D*_x_ = 1.320 Mg m^−^^3^
Hall symbol: -P 1	Mo *K*α radiation, λ = 0.71073 Å
*a* = 7.4220 (4) Å	Cell parameters from 7401 reflections
*b* = 9.7120 (5) Å	θ = 2.5–28.2°
*c* = 11.6331 (6) Å	µ = 0.14 mm^−^^1^
α = 112.517 (2)°	*T* = 296 K
β = 94.824 (3)°	Block, yellow
γ = 109.574 (2)°	0.48 × 0.25 × 0.25 mm
*V* = 707.79 (7) Å^3^	

### Data collection

**Table:**

Bruker APEXII CCD diffractometer	3405 independent reflections
Radiation source: fine-focus sealed tube	2923 reflections with *I* > 2σ(*I*)
graphite	*R*_int_ = 0.017
φ and ω scans	θ_max_ = 28.2°, θ_min_ = 2.0°
Absorption correction: multi-scan (*SADABS*; Bruker, 2008)	*h* = −9→8
*T*_min_ = 0.937, *T*_max_ = 0.966	*k* = −12→12
11757 measured reflections	*l* = −14→15

### Refinement

**Table:**

Refinement on *F*^2^	Primary atom site location: structure-invariant direct methods
Least-squares matrix: full	Secondary atom site location: difference Fourier map
*R*[*F*^2^ > 2σ(*F*^2^)] = 0.039	Hydrogen site location: inferred from neighbouring sites
*wR*(*F*^2^) = 0.122	H-atom parameters constrained
*S* = 1.03	*w* = 1/[σ^2^(*F*_o_^2^) + (0.0679*P*)^2^ + 0.1915*P*] where *P* = (*F*_o_^2^ + 2*F*_c_^2^)/3
3405 reflections	(Δ/σ)_max_ = 0.001
182 parameters	Δρ_max_ = 0.28 e Å^−^^3^
0 restraints	Δρ_min_ = −0.26 e Å^−^^3^

### Special details

**Table:**

Geometry. All s.u.'s (except the s.u. in the dihedral angle between two l.s. planes) are estimated using the full covariance matrix. The cell s.u.'s are taken into account individually in the estimation of s.u.'s in distances, angles and torsion angles; correlations between s.u.'s in cell parameters are only used when they are defined by crystal symmetry. An approximate (isotropic) treatment of cell s.u.'s is used for estimating s.u.'s involving l.s. planes.
Refinement. Refinement of F^2^ against ALL reflections. The weighted R-factor wR and goodness of fit S are based on F^2^, conventional R-factors R are based on F, with F set to zero for negative F^2^. The threshold expression of F^2^ > 2σ(F^2^) is used only for calculating R-factors(gt) etc. and is not relevant to the choice of reflections for refinement. R-factors based on F^2^ are statistically about twice as large as those based on F, and R-factors based on ALL data will be even larger.

### Fractional atomic coordinates and isotropic or equivalent isotropic displacement parameters (Å^2^)

**Table:**

	*x*	*y*	*z*	*U*_iso_*/*U*_eq_	
Al	0.05537 (5)	0.05266 (4)	0.89433 (3)	0.03495 (10)	
O	0.05448 (13)	0.14683 (10)	1.06417 (8)	0.0383 (2)	
N1	0.16914 (16)	0.28879 (13)	0.90600 (11)	0.0394 (2)	
N2	0.20710 (15)	0.42899 (12)	1.00733 (10)	0.0366 (2)	
N3	0.28396 (18)	0.56192 (13)	0.98973 (12)	0.0457 (3)	
C1	0.10477 (17)	0.30216 (14)	1.15315 (12)	0.0347 (3)	
C2	0.17660 (18)	0.44012 (14)	1.12950 (12)	0.0348 (3)	
C3	0.2281 (2)	0.59650 (15)	1.22664 (13)	0.0420 (3)	
H3B	0.2737	0.6849	1.2076	0.050*	
C4	0.2129 (2)	0.62320 (16)	1.35011 (13)	0.0435 (3)	
C5	0.1424 (2)	0.48768 (17)	1.37431 (13)	0.0432 (3)	
H5A	0.1312	0.5018	1.4567	0.052*	
C6	0.0886 (2)	0.33235 (16)	1.27846 (13)	0.0421 (3)	
H6A	0.0399	0.2447	1.2982	0.051*	
C7	0.22611 (19)	0.33415 (16)	0.81377 (13)	0.0409 (3)	
C8	0.2240 (2)	0.2418 (2)	0.68526 (15)	0.0546 (4)	
H8A	0.1766	0.1290	0.6486	0.065*	
C9	0.2959 (2)	0.3281 (2)	0.61786 (16)	0.0588 (4)	
H9A	0.2961	0.2715	0.5328	0.071*	
C10	0.3694 (2)	0.4988 (2)	0.67200 (16)	0.0566 (4)	
H10A	0.4178	0.5511	0.6219	0.068*	
C11	0.3719 (2)	0.5895 (2)	0.79461 (16)	0.0534 (4)	
H11A	0.4203	0.7023	0.8297	0.064*	
C12	0.29715 (19)	0.50377 (16)	0.86672 (14)	0.0416 (3)	
C13	0.2747 (3)	0.7923 (2)	1.45629 (17)	0.0637 (5)	
H13A	0.3229	0.8706	1.4231	0.096*	
H13B	0.3771	0.8112	1.5237	0.096*	
H13C	0.1635	0.8026	1.4896	0.096*	
C14	−0.1914 (2)	−0.00914 (18)	0.77213 (15)	0.0490 (3)	
H14A	−0.2766	−0.1197	0.7492	0.073*	
H14B	−0.1613	0.0013	0.6964	0.073*	
H14C	−0.2562	0.0610	0.8114	0.073*	
C15	0.2981 (2)	0.02143 (18)	0.86005 (16)	0.0501 (3)	
H15A	0.2845	−0.0846	0.8501	0.075*	
H15B	0.4081	0.1025	0.9306	0.075*	
H15C	0.3199	0.0312	0.7828	0.075*	

### Atomic displacement parameters (Å^2^)

**Table:**

	*U*^11^	*U*^22^	*U*^33^	*U*^12^	*U*^13^	*U*^23^
Al	0.03920 (19)	0.03075 (17)	0.03427 (18)	0.01356 (14)	0.01205 (14)	0.01332 (13)
O	0.0501 (5)	0.0270 (4)	0.0362 (4)	0.0132 (3)	0.0149 (4)	0.0131 (3)
N1	0.0451 (5)	0.0326 (5)	0.0389 (5)	0.0130 (4)	0.0134 (4)	0.0155 (4)
N2	0.0380 (5)	0.0293 (4)	0.0405 (5)	0.0110 (4)	0.0075 (4)	0.0160 (4)
N3	0.0533 (6)	0.0335 (5)	0.0504 (6)	0.0124 (5)	0.0101 (5)	0.0231 (5)
C1	0.0349 (5)	0.0294 (5)	0.0369 (6)	0.0121 (4)	0.0098 (4)	0.0122 (4)
C2	0.0354 (5)	0.0312 (5)	0.0362 (6)	0.0136 (4)	0.0064 (4)	0.0132 (4)
C3	0.0457 (7)	0.0305 (5)	0.0440 (7)	0.0144 (5)	0.0041 (5)	0.0128 (5)
C4	0.0436 (6)	0.0354 (6)	0.0414 (7)	0.0166 (5)	0.0031 (5)	0.0074 (5)
C5	0.0450 (7)	0.0433 (6)	0.0353 (6)	0.0178 (5)	0.0108 (5)	0.0110 (5)
C6	0.0473 (7)	0.0382 (6)	0.0403 (6)	0.0158 (5)	0.0156 (5)	0.0167 (5)
C7	0.0398 (6)	0.0427 (6)	0.0441 (6)	0.0150 (5)	0.0134 (5)	0.0234 (5)
C8	0.0635 (9)	0.0543 (8)	0.0464 (7)	0.0219 (7)	0.0219 (7)	0.0223 (6)
C9	0.0581 (8)	0.0775 (10)	0.0476 (8)	0.0259 (8)	0.0207 (6)	0.0338 (7)
C10	0.0463 (7)	0.0758 (9)	0.0619 (9)	0.0192 (7)	0.0156 (6)	0.0483 (7)
C11	0.0506 (8)	0.0551 (7)	0.0636 (9)	0.0157 (6)	0.0132 (6)	0.0395 (7)
C12	0.0382 (6)	0.0427 (6)	0.0481 (7)	0.0141 (5)	0.0086 (5)	0.0261 (5)
C13	0.0802 (11)	0.0409 (8)	0.0481 (9)	0.0224 (8)	0.0030 (8)	0.0019 (6)
C14	0.0484 (7)	0.0451 (7)	0.0506 (8)	0.0163 (6)	0.0066 (6)	0.0213 (6)
C15	0.0452 (7)	0.0443 (7)	0.0595 (8)	0.0189 (6)	0.0192 (6)	0.0192 (6)

### Geometric parameters (Å, °)

**Table:**

Al—O	1.8337 (10)	C6—H6A	0.9300
Al—C15	1.9725 (15)	C7—C12	1.3991 (19)
Al—C14	1.9767 (15)	C7—C8	1.414 (2)
Al—O^i^	2.0918 (10)	C8—C9	1.370 (2)
Al—N1	2.1060 (11)	C8—H8A	0.9300
O—C1	1.3595 (14)	C9—C10	1.407 (2)
O—Al^i^	2.0918 (10)	C9—H9A	0.9300
N1—N2	1.3344 (15)	C10—C11	1.354 (2)
N1—C7	1.3556 (18)	C10—H10A	0.9300
N2—N3	1.3277 (15)	C11—C12	1.417 (2)
N2—C2	1.4268 (17)	C11—H11A	0.9300
N3—C12	1.3483 (19)	C13—H13A	0.9600
C1—C6	1.3967 (18)	C13—H13B	0.9600
C1—C2	1.4084 (18)	C13—H13C	0.9600
C2—C3	1.3978 (17)	C14—H14A	0.9600
C3—C4	1.380 (2)	C14—H14B	0.9600
C3—H3B	0.9300	C14—H14C	0.9600
C4—C5	1.390 (2)	C15—H15A	0.9600
C4—C13	1.5098 (19)	C15—H15B	0.9600
C5—C6	1.3826 (18)	C15—H15C	0.9600
C5—H5A	0.9300		
			
O—Al—C15	115.55 (6)	C1—C6—H6A	118.8
O—Al—C14	114.97 (6)	N1—C7—C12	107.66 (12)
C15—Al—C14	129.44 (7)	N1—C7—C8	131.38 (13)
O—Al—O^i^	76.96 (4)	C12—C7—C8	120.97 (13)
C15—Al—O^i^	94.55 (6)	C9—C8—C7	116.14 (15)
C14—Al—O^i^	94.85 (5)	C9—C8—H8A	121.9
O—Al—N1	87.28 (4)	C7—C8—H8A	121.9
C15—Al—N1	92.11 (6)	C8—C9—C10	122.70 (16)
C14—Al—N1	91.89 (6)	C8—C9—H9A	118.7
O^i^—Al—N1	164.24 (4)	C10—C9—H9A	118.7
C1—O—Al	134.46 (8)	C11—C10—C9	122.11 (15)
C1—O—Al^i^	122.50 (8)	C11—C10—H10A	118.9
Al—O—Al^i^	103.04 (4)	C9—C10—H10A	118.9
N2—N1—C7	103.95 (11)	C10—C11—C12	116.60 (15)
N2—N1—Al	127.89 (9)	C10—C11—H11A	121.7
C7—N1—Al	128.13 (9)	C12—C11—H11A	121.7
N3—N2—N1	115.67 (11)	N3—C12—C7	109.19 (12)
N3—N2—C2	120.79 (11)	N3—C12—C11	129.32 (13)
N1—N2—C2	123.49 (10)	C7—C12—C11	121.48 (14)
N2—N3—C12	103.53 (11)	C4—C13—H13A	109.5
O—C1—C6	119.56 (11)	C4—C13—H13B	109.5
O—C1—C2	124.65 (11)	H13A—C13—H13B	109.5
C6—C1—C2	115.79 (11)	C4—C13—H13C	109.5
C3—C2—C1	121.32 (12)	H13A—C13—H13C	109.5
C3—C2—N2	116.43 (11)	H13B—C13—H13C	109.5
C1—C2—N2	122.22 (11)	Al—C14—H14A	109.5
C4—C3—C2	121.75 (13)	Al—C14—H14B	109.5
C4—C3—H3B	119.1	H14A—C14—H14B	109.5
C2—C3—H3B	119.1	Al—C14—H14C	109.5
C3—C4—C5	117.29 (12)	H14A—C14—H14C	109.5
C3—C4—C13	121.89 (14)	H14B—C14—H14C	109.5
C5—C4—C13	120.80 (14)	Al—C15—H15A	109.5
C6—C5—C4	121.39 (13)	Al—C15—H15B	109.5
C6—C5—H5A	119.3	H15A—C15—H15B	109.5
C4—C5—H5A	119.3	Al—C15—H15C	109.5
C5—C6—C1	122.45 (13)	H15A—C15—H15C	109.5
C5—C6—H6A	118.8	H15B—C15—H15C	109.5

Symmetry codes: (i) −*x*, −*y*, −*z*+2.
